# The future of medicine: an outline attempt using state-of-the-art business and scientific trends

**DOI:** 10.3389/fmed.2024.1391727

**Published:** 2024-08-07

**Authors:** Gregorios Agyralides

**Affiliations:** Medical Division, Boehringer Ingelheim Hellas Single Member S.A., Kallithea, Greece

**Keywords:** future of medicines, pharma ecosystem, artificial intelligence, nanotechnology, artificial neural networks, personalized medicine, machine learning, real-world data

## Abstract

**Introduction:**

Currently, there is a lot of discussion about the future of medicine. From research and development to regulatory approval and access to patients until the withdrawal of a medicinal product from the market, there have been many challenges and a lot of barriers to overcome. In parallel, the business environment changes rapidly. So, the big question is how the pharma ecosystem will evolve in the future.

**Methods:**

The current literature about the latest business and scientific evolutions and trends was reviewed.

**Results:**

In the business environment, vast changes have taken place via the development of the internet as well as the Internet of Things. A new approach to production has emerged in a frame called Creative Commons; producer and consumer may be gradually identified in the context of the same process. As technology rapidly evolves, it is dominated by Artificial Intelligence (AI), its subset, Machine Learning, and the use of Big Data and Real-World Data (RWD) to produce Real-World Evidence (RWE). Nanotechnology is an inter-science field that gives new opportunities for the manufacturing of devices and products that have dimensions of a billionth of a meter. Artificial Neural Networks and Deep Learning (DL) are mimicking the use of the human brain, combining computer science with new theoretical foundations for complex systems. The implementation of these evolutions has already been initiated in the medicinal products’ lifecycle, including screening of drug candidates, clinical trials, pharmacovigilance (PV), marketing authorization, manufacturing, and the supply chain. This has emerged as a new ecosystem which features characteristics such as free online tools and free data available online. Personalized medicine is a breakthrough field where tailor-made therapeutic solutions can be provided customized to the genome of each patient.

**Conclusion:**

Various interactions take place as the pharma ecosystem and technology rapidly evolve. This can lead to better, safer, and more effective treatments that are developed faster and with a more solid, data-driven and evidence-concrete approach, which will drive the benefit for the patient.

## 1 Introduction

In recent years, a lot of discussion has been ongoing with regard to the next day in medicines. Short-term as well as long-term plans for the future of the pharmaceutical sector are outlined by the relevant stakeholders, such as the pharma industry, regulators, and payers. Pharmaceutical technology is rapidly evolving, spearheaded by brand-new dosage forms that allow optimal drug targeting and treatment individualization for patients. In terms of the business environment, vast changes have taken place during the last 25 years bringing to light new types of business practices. This study attempts to go one step beyond and outline a possible future for the medicinal sector, considering current facts and trends as well as brand-new and innovative approaches in both the business and the pharmaceutical area.

## 2 Methods

This study was based on a review of the literature. With regard to the latest scientific evolutions and trends, we focused on the incorporation of pharmaceutics’ state-of-the-art technologies. PubMed was used as the main source of information. The keywords used were: artificial intelligence, nanotechnology, big data, real-world data (RWD), and real-world evidence (RWE). When considering that further research is necessary, further keywords were used based on the initial research: artificial neural networks, machine learning, and deep learning. Articles published as recent as 2019 were preferred, although, optionally, older articles were used upon consideration that they shed some more light on the study. With regard to health authorities and regulatory-relevant topics, the websites of the relevant authorities and agency bodies of the USA and Europe, such as the US Food and Drug Administration (FDA), European Medicines Agency (EMA), and Heads of Medicines Agencies (HMA), were used as sources of information. The book “Modern Pharmaceutical Nanotechnology: Basic Principles and Practical Applications” by Professor K.N. Demetzos was also used as a source since it is considered state-of-the-art in the pertinent scientific area. Please refer to Table 1.

**Table 1:** Literature search methodology followed for scientific evolutions.

**Scientific evolutions**
Source: PubMed
**First level search keywords:**
Artificial intelligence, nanotechnology, big data, real-world data, real-world evidence
**Second level search keywords:**
Artificial neural networks, machine learning, deep learning
**Regulatory environment search:**
Websites of the relevant authorities and agency bodies in the USA and Europe (e.g., FDA, EMA, and HMA)
**Additional source:**
“Modern Pharmaceutical Nanotechnology: Basic Principles and Practical Applications” by Professor K.N. Demetzos

With regard to the business environment, as a starting point, the book “Post-capitalism: a guide to our future” by Paul Mason was used, as it was considered to provide a robust and highly interesting overview of the current situation and appropriately links the past, the present and the future, so it was considered a challenge to combine it with the pharmaceutical sector in a concurrent research project. Based on this source, supporting literature that is used by it was further researched, and if judged appropriate, it was referred to, e.g., Peter Drucker’s theory (refer to Table 2 and Section Limitations).

**Table 2:** Literature search methodology followed for the business environment.

**Business environment**		
“Post-capitalism: a guide to our future” by Paul Mason		
↓	↓	↓
Post-Capitalist Society by P. Drucker	The Wealth of Networks: How Social Production Transforms Markets and Freedom by Y. Benkler	Did Hilferding Influence Schumpeter? by P. Michailides and J. Milios

## 3 Results

### 3.1 Internet-driven environment and creative commons

Currently, the driving force of innovation is not the isolated inventor in his/her laboratory but the big corporations that make use of the capabilities of applied sciences (1). Already in 1993, the renowned business management theory guru Peter Drucker wrote that knowledge is now the main resource and not one resource. Drucker focused on the training methods of employees in fields where knowledge is necessary to develop a combinatory way of thinking of the mind like Einstein’s. Drucker was based on an appropriate methodology and a well-designed schedule that would optimize the training and education of employees. Connection and vertebrate use of information would be the key factors in increasing productivity. However, humanity found a better answer: the internet. It was neither a product of a central design nor an administrative program, but the outcome of the spontaneous interaction of people using web routes and forms of connection that did not exist 30 years ago. That contributes to what Drucker called “globally educated humans” (2). The internet provides a type of socialized economy. In 1997, only 2% of the world’s population had access to the internet. Now this percentage has increased to 67% (3).

Since 2004, after the internet dominated with portable means, technology has boosted a new type of entrepreneurship: Web 2.0. In 2006, Yochai Benkler, a law professor at Yale University, concluded that the internet offers a new mode of production rising in the heart of the most developed economies. Benkler described standards of common use of products and practices of non-hierarchical production. He called this frame Creative Commons. First and foremost, the introduction of cheap computing power as well as communication networks gave a huge number of people the means to produce intellectual products. Today, everyone can have a blog, create and distribute movies, and self-publish e-books, sometimes obtaining a public of millions before the author’s name reaches the traditional publishing houses (4). If something can be copied and pasted, it can be freely reproduced with zero cost. Wikipedia is the best example: a digital encyclopedia established in 2001, it was authored collectively, and now it contains 26 million entries. Approximately 24 million people have been available either to contribute with texts or to correct the existing ones. Regular scribers are 12,000 and circumstantial 140,000. The personnel of Wikipedia are only 208. All the scribers offer their text without any financial profit. Wikipedia does not make any profit. However, the most important thing is that it is now one of the most useful information resources. In such an environment, producer and consumer are gradually identified in the same process (5).

It is expected that in the next 10 years, billions of connections between devices will be established. Known as the Internet of Things (IoT), it will colonize the worldwide net of information with so many smart machines that will overrule numerically the planet’s population. When knowledge becomes social in the context of collective intelligence, a part of value occurs for free, as follows (5):

- Informational goods leverage general scientific knowledge.
- Users are fed back in real-time with data that enhance optimization at no cost.
- Every evolution in knowledge, in whichever place it may appear, can be immediately applied to any device.

Refer Table 3.

**Table 3:** Internet-driven environment and creative commons.

**Internet-driven environment and creative commons**			
Knowledge →	Information →	Internet →	Globally educated human
↓↑			
Creative commons			
↓↑			
Internet of things			

### 3.2 Technology evolution

#### 3.2.1 Artificial intelligence

Humans have always dreamed of “recreating” human intelligence in machines. Nowadays, this has come true to a high degree, as many information system groups are developing learning algorithms to “mimic” human learning and decision-making. This is what is described as Artificial Intelligence [AI; (6)].

The promising possibilities of AI are being extensively described lately. More precisely, AI is defined as “the part of computer science concerned with designing systems that exhibit the characteristics we associate with intelligence in human behavior” (7). In the context of AI, Machine Learning (ML) involves the implementation of algorithms and statistical models to improve its performance without explicit programming; this requires the use of large amounts of data and allows the creation of patterns and relationships in these data (8). Furthermore, open AI has developed a very strong processing model, GPT-3, with the potential to generate human-like text on demand (9). Generative AI is a form of ML that can generate data in various formats (such as text, image, audio, video, or code) and adapt to new tasks in real-time based on simple text-based prompts. This renders generative AI flexible since one “model” (e.g., DALL-E or ChatGPT) can be used for various tasks (including medical research). Generative AI could be used as a tool for researchers during all stages of the research pipeline, making the entire process more cost-effective and efficient (10).

#### 3.2.2 Big data, real-world data, and real-world evidence

To better understand this section, we need to start with some definitions. The arm of science that is called Data Science is defined as “the set of fundamental principles that support and guide the principled extraction of information and knowledge from data.” Big Data are defined as “digital data that are generated in high volume and variety and that accumulate at high velocity, resulting in datasets too large for traditional data-processing systems” (11). Big Data presents many opportunities for translational studies, setting informatics at the center of successful translational research. This can revolutionize healthcare using large-scale measurements of individuals (12). RWD are data relating to patient health status and/or the delivery of healthcare routinely collected from a variety of sources (13).

The goal of the use of RWD is to generate evidence. The term Real-World Evidence (RWE) is highly related to RWD. Focusing on healthcare, there have been various definitions of RWE. The definition provided by the British Medicines and Healthcare Products Regulatory Agency (MHRA) is “information produced from data routinely collected on patients, such as Electronic Health Records (EHR) as well as disease and patient registries,” while the US FDA uses “clinical evidence regarding the usage and potential benefits or risks of a medical product derived from analysis of RWD.” In parallel, the US Congress defines RWD utilized in RWE as “data regarding the usage, or the potential benefits or risks, of a drug derived from sources other than traditional clinical trials” (14). RWE-based research makes use of various sources, such as laboratory data, hospital data, claims and billing activities in EHRs, drug and disease registries, patient-generated data, including in home-use settings, and other data from several sources related to health conditions, such as mobile devices, as well as approaches to linking these data. The approach of RWD is different from the collection of data in an experimental setting such as a Randomized Controlled Trial (RCT), as they are mainly observational data (15).

#### 3.2.3 Nanotechnology

Nanotechnology is an inter-science field pertaining to the development and use of materials for the manufacturing of devices and products that have dimensions of a billionth of a meter. Nanoparticles and nanomaterials feature new properties that are related to their dimensions. The term nanotechnology embraces the science and technology where the structural units of matter are developed at the nanoscale for the development of complex macromolecular systems, while new properties of materials appear due to this escalation as they evolve (16). Nanomedicine, or nanotechnology-based medicine, refers to the characterization of surface properties and the design of nanocarriers for several medical strategies (Table 4).

**Table 4:** Technology evolution, description, application in medicine development and relevant therapeutic uses.

Technology	Description	Examples	Application in medicine development	Therapeutic use
Artificial intelligence	The part of computer science concerned with designing systems that exhibit the characteristics we associate with intelligence in human behavior	GPT-3 IBM’s Watson, AdmetSAR 2.0, and ADMETLab 2.0 FP-ADMET Interpretable-ADMET HelixADMET Atomic Healthentia CRISPR	Non-clinical research Screening drug candidates Customize medicines for individual patients Pharmacokinetics studies Clinical Trials GPT-3 can generate a GCP-relevant electronic Case Report Form (eCRF) from a protocol. GPT-3 can facilitate the creation of multiple applications or protocol amendments much faster. Pharmacovigilance signal identification and handling Drug–drug interactions Shortage Monitoring Monitor and predict epidemic outbreaks or seasonal diseases Pediatric trials Simulation of clinical trials PV data receipt and processing	Cancer Cardiovascular Diseases Neural diseases Sickle cell anemia Beta-thalassemia
Machine learning	A subset of AI that involves the implementation of algorithms and statistical models to improve its performance without explicit programming	Generative AI DALL-E ChatGPT CRISPR	Tool for researchers during all stages of the research pipeline, making the entire process more cost-effective and efficient. In clinical development, it supports electronic study data monitoring, and thus ensures the correctness of the data and patient safety, while also ensuring cost-effectiveness and clinical trial simulation Pharmacovigilance signal identification and handling Drug–drug interactions Shortage Monitoring Monitor and predict epidemic outbreaks or seasonal diseases Simulation of clinical trials PV data receipt and processing PV disproportionality analysis PV and pharmaepidemiology predictive models Screening social media for Adverse Events (AEs) Identify topics of polypharmacy and patient diversity Safety assessments from the pre-marketing to the post-marketing phase	Sickle cell anemia Beta-thalassemia
Deep learning	A subset of ANNs that incorporates multiple layers of hidden layers of artificial neural networks		Creation of end-to-end predictive models	Nodular BCC Dermal nevus and seborrheic keratosis in dermatopathology and diabetic retinopathy Type 2 diabetes subgroups, Cytological abnormalities in females Cardiac anomalies and congestive heart failure
Big data	Digital data that are generated in high volume and high variety and that accumulate at high velocity, resulting in datasets too large for traditional data-processing systems		Translational studies, setting informatics to the center of successful translational research	
Real-world data	Data relating to patient health status and/or the delivery of healthcare is routinely collected from a variety of sources Mainly observational data	Laboratory data, hospital data, claims and billing activities, EHRs, drug and disease registries, patient-generated data including in home-use settings, and other data from several sources related to health conditions such as mobile devices as well as approaches to link these data	Generating evidence External control arm in Clinical Trials Enable Healthcare decision-making Better planning and conduct of clinical trials Meet unmet medical needs Simulation of clinical trials	Epilepsy Oncology Lung Cancer
Real-world evidence	Clinical evidence regarding the usage and potential benefits or risks of a medical product derived from analysis of RWD	Information produced from data routinely collected on patients, such as Electronic Health Records (EHR) as well as disease and patient registries	Generating Data for Marketing Authorization Applications Enable first and post-approval processes Enable Healthcare decision-making Better planning and conduct of clinical trials Meet unmet medical needs	COVID-19 vaccine, Oncology Lung Cancer Orphan Drugs
Nanotechnology	The inter-science field pertaining to the development and use of materials for the manufacturing of devices and products that have dimensions of a billionth of a meter	Nanomedicine or Nanotechnology-based medicine	Design of nanocarriers for several medical strategies	CNS diseases
		Pharmaceutical Nanotechnology	Targeted and controlled release customized in the anatomy of each patient Optimization of diagnosis, e.g., in case of epileptic seizures	
		Nanotheranostics	Facilitate the profiling of disease and medicine, as well as a deeper understanding of the relationship between host and disease Detection and recognition of pathological signs which can lead to the creation of databases with information for further clinical	
Artificial neural networks	The outcome of the combination of computer science with new theoretical foundations for complex systems. ANNs are representation-learning algorithms composed of three processing layers with a finite number of nonlinear units, called artificial neurons		Quality by Design models Continuous Manufacturing Bioequivalence studies	Sjögren’s disease Restorative Dentistry Endodontics, Orthodontics Dental Surgery and Periodontology
Precision medicine	Provision of tailored healthcare to patients with lower rates of associated adverse outcomes		More precise approach for the prevention, diagnosis, and treatment of disease “4Ps”—Predictive, Preventive, Personalized, and Participatory, while recently a fifth “P” has been added, standing for Population-wise Perspective Tailored healthcare to patients with lower rates of associated adverse outcomes Amend the disease-associated organelles	Cancer Neural diseases Cardiovascular diseases HER2-positive breast cancer Nodular BCC Dermal nevus and seborrheic keratosis in Dermatopathology, Diabetic retinopathy Type 2 diabetes subgroups, Cytological abnormalities in females Cardiac anomalies, Congestive heart failure
Personalized medicine	A targeted approach to the prevention, diagnosis and treatment of disease based on an individual’s specific profile		Better disease management assessment Primarily based on disease-related clinical, genomic, metabolomics and environmental information and is a broad and rapidly advancing field in healthcare Proactive intervention and clinical decision support system for better predictions on the potential outcome of patients, ultimately supporting better decision-making by the physicians Amend the disease-associated organelles	Cancer Neural diseases Cardiovascular diseases HER2-positive breast cancer Nodular BCC dermal nevus and seborrheic keratosis in dermatopathology, diabetic retinopathy type 2 diabetes subgroups, cytological abnormalities in females cardiac anomalies, congestive heart failure
Drug targeting	Direct targeting of drugs into subcellular compartments		Optimizes therapeutic efficacy and safety of medicines	Cancer, viral diseases
Gene therapy	Medicines produced by cloning a normal human gene		Treat or demonstrate disease stabilization	Genetic disorders Infectious and genetic diseases Retroviral diseases Neurodegenerative diseases

Pharmaceutical Nanotechnology can optimize diagnosis processes via the use of nanoscale biosensors that record the electrical excitability of the brain. Based on these data, algorithms can be developed to make predictions or characterizations of clinical conditions such as epileptic seizures, including their socio-economic impact.

Nano-therapeutic systems used for diagnostic purposes are defined as nanotheranostics. In combination with IoT and AI, they bring new potential as they allow the detection and recognition of pathological signs, which can lead to the creation of databases with information for further clinical use (17).

#### 3.2.4 Artificial neural networks

Artificial Neural Networks (ANNs) are the outcome of the combination of computer science with new theoretical foundations for complex systems. ANNs are representation-learning algorithms composed of three processing layers with a finite number of nonlinear units, called artificial neurons. The first and third layers are defined as input and output layers, respectively. The second layer contains a variable number of artificial neurons called hidden units, which compute the nonlinearity of a given problem. By using a set of simple rules, ANNs can learn from some of the data and apply their “knowledge” to the rest of the available information. ANN’s main feature is the ability to modify its internal structure in response to the available data. In comparison with the typical statistical methods used in epidemiology, these models can analyze all signals in parallel and estimate nonlinear relationships between all the variables considered. Similarly, with the human brain, ANNs can recognize patterns, use data, and learn by example, as a healthcare professional does in daily practice.

A subset of ANNs that incorporates multiple layers of hidden layers of artificial neural networks is Deep Learning [DL; (18)]. DL can process raw data like those contained in digital images and can create end-to-end predictive models by performing all the processing steps usually involved in the design of a classic ANN model, including feature extraction and learning. The multi-layered structure of deep neural networks allows them to serve as nonlinear function approximators, able to learn different representations of the input data at multiple levels of abstraction (19).

### 3.3 Applications in pharmaceutics’ lifecycle

#### 3.3.1 Screening drug candidates

The discovery, development, and launch of a novel drug to market is a hard, long, and complicated process that includes the following steps: drug discovery, preclinical studies, clinical trials, and regulatory approval. It appears that only 7.9% of total drug candidates successfully make it to the market. One of the most crucial steps to improving drug development success rates is to choose the best lead drug candidate. Several advances have recently taken place concerning *in silico* research, which has enabled a drastic reduction in the time and cost of screening undesired drug candidates. Prediction of a drug’s failure by 10% before a drug candidate enters clinical trials could save approximately USD 100 million in development expenses for each drug (8).

AI-based drug research and development appear to be a promising factor and a large force in the pharmaceutical area, and it is expected to make significant improvements in non-clinical research. The vast success of AI is, among others, that AI algorithms can predict many properties of a compound *in silico* rather than *in vitro* or *in vivo* (20). During drug development, AI enables the initial screening of drug compounds via a predictable success rate based on biological factors and rapid measurement of Ribonucleic Acid (RNA) and Deoxyribonucleic Acid (DNA) by sequencing. Next-generation sequencing allows the fast discovery of drugs and customized medicines for individual patients. Ranging from the development of new molecules to the identification of new biological targets, AI plays a significant role in drug identification, validation, drug discovery based on targeted, phenotypic, and multipurpose drugs, drug reassignment, and the identification of biomarkers (21). As an example, IBM’s Watson identifies connections and relationships among genes, drugs, diseases, and other entities by analyzing multiple data sets of life sciences knowledge (9).

Furthermore, the identification of chemical candidates with higher efficacy and lower toxicity becomes a major challenge. This includes pharmacokinetics studies, i.e., absorption, distribution, metabolism, excretion, and toxicity (ADMET) for a candidate drug. The drug distribution process can be evaluated, and factors such as Blood–Brain Barrier Permeability, Plasma Protein Binding, Fraction Unbound in Plasma, and Volume of Distribution can be predicted. Most importantly, this can take place with the use of a list of free tools and available databases and datasets that are offered to the scientific community for free. Efficient AI-based prediction tools for diverse endpoints have been developed. For example, five publicly available ADMET predictors were developed between 2019 and 2022: AdmetSAR 2.0, ADMETLab 2.0, FP-ADMET, Interpretable-ADMET, and Helix-ADMET. They easily identify ADMET profiles for a wide range of drug candidates, and they are available for free. On the other hand, an AI-based predictive model depends highly on the data and the modeling approach. An increasing number of public datasets, which facilitate the collection of many structural substances and their associated experimental values, are now available. Some of the most popular data sources for AI-based ADMET prediction research are ZINC20, ChemSpider, PubChem, Therapeutics Data Commons and OCHEM 4.2. It seems that the further evolution of big data will lead to prospects for future non-clinical drug research (8).

#### 3.3.2 Clinical trials

A popular area of AI implementation is the field of clinical trials. It has been calculated that only 13.8% of drugs successfully pass clinical trials. A pharma company is expected to spend USD 1.3 billion on average for any drug to complete the entire clinical trial process and receive the health authorities’ approval. The potential of AI is to efficiently transform the key stages of clinical trial development from study design to implementation to ensure or increase the probability of trial success while simultaneously reducing the workload of scientific personnel. AI algorithms can automatically identify suitable study candidates by evaluating parameters like history and disease conditions, as well as additional characteristics like infection rate, demographics, and ethnicity. In this context, RWD and RWE play an important role in healthcare decision-making. To collect and store large amounts of health-related data, the use of computers, mobile devices, wearables, and other biosensors is rapidly accelerating. Processing these data can lead to better planning and conduct of clinical trials and possibly meet unmet medical needs (22).

Furthermore, AI can speed up the development of clinical trials and provide an early warning system in case trials do not produce substantial results; data validity, completeness, representativeness, and timeliness can be assessed. RWD-based trials via the use of AI can profile patients in a study (e.g., via the use of DNA sequencing, metabolomics, proteomics, etc.) and match the study drugs with the clinical conditions identified in the profiling. AI analysis of large relevant datasets can help this process, as can ML, which can support electronic study data monitoring and thus ensure the correctness of the data and patient safety, while also ensuring cost-effectiveness. A more complete picture of drug discovery and biomarkers can be obtained via other types of RWD, such as genomics and patient-reported issues, in combination with EHR data and AI and ML techniques. GPT-3 has been proposed to be used in clinical trial processes for tasks like generating a GCP-relevant electronic Case Report Form (eCRF) from a protocol. GPT-3 could even facilitate the creation of multiple applications or protocol amendments much faster (23).

Simulation of clinical trials is a very challenging area; AI can also play a highly important role. Through the creation of external control arms with the use of ML, several scenarios can be simulated. Both drug safety and data bias can be assessed when using RWD for clinical trial simulation. ML, in combination with RWD, has been used for the simulation of colorectal cancer clinical trials focusing on drug safety. The risk ratios of serious adverse events calculated from simulations comparing two treatment arms were similar to the risk ratios measured from the trials, proving that ML and RWD can be used for the simulation of clinical trials (6).

#### 3.3.3 Pharmacovigilance

One of the main characteristics of Pharmacovigilance (PV) is that its data derive from various sources, creating a significant heterogeneity: various types of medicines, including vaccines. Thus, adverse drug reactions (ADRs) and adverse drug effects (ADEs) are manifested in several ways, and their mechanisms are of various causes and often unknown. Thus, the pertinent data recording cannot be considered solid. Analyzing this amount of data is inevitably challenging. It has been shown that AI/ML can enhance multiple aspects of data receipt and processing, including duplicate reports, harmonize identification, ensure quality assurance and assess causality relationships in individual case safety reports (ICSRs). Since the late 1990s, basic ML has been routinely applied as association rule analysis dealing with disproportionality analysis in PV to create alerts of specific drug/vaccine event pairs that have to undergo clinical review. In addition, ML has been used to identify complicated patterns in assessing PV data and for the creation of predictive models, as well as in pharmacoepidemiology. Screening social media posts, browsing electronic medical records for adverse event/reaction reporting, and accumulating data from various sources with multiple analytical approaches can ensure information-rich data insights (7).

Using ML, safety signals can be identified via automated processes and training. Thus, adverse drug events/reactions can be detected, and a safety surveillance overview can be performed. While it may be difficult for medical scientists to detect ADE/ADRs in social media, ML applications can enable the automation of classification of first-person reports of ADRs in social media (e.g., X, formerly Twitter) via identification of micro-blog messages (“tweets”) that describe spontaneous individual patient experiences, enhancing post-marketing pharmacovigilance (PV) (24). Various factors can be implemented in the process to identify topics such as polypharmacy and patient diversity (25). Another method is the use of a reference standard to compare rule-based queries with semi-supervised ML to create safety assessments from the pre-marketing to the post-marketing phase. A large amount of data concerning the side effects of therapies can be analyzed, which plays an important role in specific diseases, such as diabetes. Since under-reporting is an important hindrance to current PV, these techniques can be significantly compromised (26).

AI and ML can be applied to define drug–drug interactions or predict the effect of a drug–drug interaction. This can have an immediate effect on improving drug safety monitoring during hospitalization (27). Furthermore, AI can identify patients at high risk for ADRs. Investigators used models of ML to form patterns of toxicity that were used to estimate the number of patients with toxicity related to fluoropyrimidine toxicity due to DPD deficiency. They concluded that this model could be applicable in clinical practice (28). Concerning the prediction of drug side effects, it has been shown that knowledge derived from literature can be combined with the spontaneous reporting system methods using downstream ML. This can highly assist in the prediction of side effects, overcoming spontaneous reporting systems’ bias in combination with under-reporting that limits the availability of data (6).

Moreover, it is for granted that AI/ML implementation in PV can reduce workload, ease complexity, and reduce costs, enabling the human factor to focus on more important aspects of patient safety (7).

#### 3.3.4 Marketing authorization

The product dossier to be compiled and submitted for regulatory approval consists of many administrative documents, as well as documents depicting the results of the conducted quality, safety, and efficacy studies. AI technology enables text creation and automation of the presentation of research results. This is also encouraged by health authorities worldwide (29). The full introduction and implementation in regulatory filings of the electronic Common Technical Dossier (eCTD) format, under the auspices of International Conference of Harmonization (ICH), initiated as early as 2003, has highly enhanced international harmonization of regulatory requirements and processes among the regions of the European Union, Japan, and the United States (30). Similar approaches are followed by other regions, while for developing countries, the Certificate of Pharmaceutical Product (CPP) under the auspices of the World Health Organization (WHO) has been implemented to facilitate the availability of new medicines and to reduce the time to market through reliance on a prior trusted analysis (31). Thus, there is a uniformity in regulatory approaches and evaluations worldwide, offering very little space for deviations in this aspect.

For decades, RWE has played a role in post-approval but not in first-approval processes. According to EMA, in marketing authorization applications submitted between 2018 and 2019, RWD was utilized up to 40%, most of them at the post-authorization phase and 18% in line extensions (i.e., extension of indication). Disease registries and hospital data were the most common sources. Implementation of RWE in early assessments of innovative therapies is expected to accelerate the approval process, particularly enabling Early Benefit Assessment. This is of utmost importance for rare diseases and orphan drugs (32). A lot of RWE results have already been implemented with regard to Marketing Authorization approval processes, with the most sound example being the COVID-19 vaccine during the recent pandemic. That included a study conducted in Israel, resulting in 94% real-world efficacy of the Pfizer/BioNTech vaccine for volunteers 1 week after their vaccination in comparison with non-vaccinated volunteers (33).

With regard to a very challenging field of today’s healthcare, oncology, in relevant new drug and biologics license applications submitted between 2015 and 2020, 11 out of 133 utilized RWE to establish safety and efficacy. In this context, real-world studies were used as external controls for supplementary efficacy data from single-arm trials in successful oncology marketing authorization procedures. The key factors of success were early engagement, *a priori* protocol development, and robust research design. A similar approach can be applied in second- or third-line treatment. As in lung cancer, there is still a lack of information on the safety and survival outcomes of immunotherapy in older adult patients over 75 years old. There has been an effort for clarification as prompted by international cancer associations and regulatory bodies to call for RCTs matching real-world situations more closely. Moreover, the inclusion of an external RWD control arm in clinical studies can also help compensate for the diversity problems of representative patients in oncology trials. Registries can play an important role, as they provide information on cancer types, population, standard-oncological treatments, and their association with high quality.

Thus, in the future, RWE can enable the enhancement of registration processes and lead to innovative therapies for patients in sensitive fields like orphan and oncological drug products (14). Moreover, with the development of sophisticated, new analytical capabilities, pharmaceutical companies can better analyze these data and apply the results of the analysis to the development and approval of medicines (9).

#### 3.3.5 Manufacturing

Innovative technologies are also a big area of application in pharmaceutical production. Quality by Design (QbD) has already been introduced in manufacturing as a methodology that assures product quality and features a well-defined roadmap. ANN based on QbD successfully support the establishment of drug specifications and constraints on process parameters, connecting formulation development to *in vitro* performance and positive clinical results derived from bioequivalence studies (34). A new approach in pharmaceutical manufacturing is Continuous Manufacturing (CM). Through the use of a Deep Neural Network (DNN) that identifies at a higher level the main critical process parameter, adequate control of the process is ensured. Synergies between process analytics and ANN application lead to a continuous production line monitoring system, which improves knowledge about this innovative production line and the products manufactured (35).

#### 3.3.6 Supply chain

A worldwide public health problem is the shortage of essential medicines. Multilayer neural networks can be used for drug shortage monitoring, allowing the forecasting apparatus to be transferred from the level of practical observations to a scientific forecasting methodology based on modern digital technologies. Modeling of the modern drug supply via analysis and forecasting is in the target state of the healthcare system. A key example of this topic is epidemic forecasting. ML and AI technologies are also being applied to monitor and predict epidemic outbreaks or seasonal diseases around the world (36). A predictive forecast can enable the planning of the supply chain to obtain inventory at the right time and in the right quantity based on the predicted accuracy (37).

### 3.4 Precision medicine, personalized medicine, and drug targeting

According to the European Alliance for Personalized Medicine (EAPM), personalized medicine is defined as “a targeted approach to the prevention, diagnosis, and treatment of disease based on an individual’s specific profile” (38). Personalized medicine is a promising field of scientific research with expectations for better disease management assessment. The US National Human Genome Research Institute defines precision medicine as: “Precision medicine (generally considered analogous to personalized medicine or individualized medicine) is an innovative approach that uses information about an individual’s genomic, environmental, and lifestyle information to guide decisions related to their medical management. The goal of precision medicine is to provide a more precise approach for the prevention, diagnosis, and treatment of disease” (39).

Precision medicine is mainly based on the “4Ps”—Predictive, Preventive, Personalized, and Participatory, while recently a fifth “P” has been added, standing for Population-wise Perspective. The primary approach of precision medicine is to provide tailored healthcare to patients with lower rates of associated adverse outcomes. Precision medicine customizes the disease treatment for a single individual in contrast with the old approach of one-size-fits-all medicine; AI and ML-based systems also play an important role as they bridge multiple domains in a secure environment for heterogeneous healthcare data analysis and visualization. Personalized medicine is primarily based on disease-related clinical, genomic, metabolomics and environmental information and is a broad and rapidly advancing field in healthcare. It incorporates the enhancement of proactive intervention through AI and clinical decision support system development to leverage big data analysis for better predictions on the potential outcome of patients, ultimately supporting better decision-making by the physicians.

Precision medicine endorses a healthcare ecosystem where clinicians and researchers obtain a complete picture of the patient using additional basic details from healthcare data, e.g., phenotypic information and social and lifestyle factors that affect treatment decisions (40).

Both precision medicine and personalized medicine can treat the diseases associated with organelles. This can be implemented in combination with drug targeting. Direct targeting of drugs into subcellular compartments is a highly promising therapeutic strategy. Drug delivery directly to specific intracellular compartments optimizes therapeutic efficacy as well as the safety of medicines. This can be achieved either indirectly via targeting the cell nucleus as the central compartment of the cell or directly via targeting the compartment itself. A tactic used for subcellular compartment targeting includes drugs or nanoshuttles labeled with the compartment’s localization signal. Another approach is the utilization of specific ligands during the design of drug delivery nanoshuttles, which facilitates the targeting of the specified intracellular compartment. Selective permeability of biomembranes, efflux transporters, and lysosomes are factors that need to be taken into consideration for the development of such drug delivery systems. Drug targeting is very promising, especially in diseases such as cancer and viral diseases (41).

## 4 Discussion

The total amount of data in our lives will soon include the speed at which we drive, our weekly diet, our cardiac pulses and our body weight, which can by itself form a particularly effective “technology of societies.” When IoT has been fully developed, the information economy will take off (5). Several key developments have led to the data revolution in healthcare: the data science movement that has transformed other industries; the extraordinary development in computational power; the availability of open-source tools and low-cost equipment for advanced analyses; and the increasing availability of educational resources and advanced degrees in data science and related fields. For training and educational purposes, there are a variety of courses from high-level institutions that cover data science and ML, many of them for free. Some websites contain user-created code to run ML algorithms from scratch (e.g., https://github.com/) or host data science competitions for participants around the world (e.g., https://www.kaggle.com/). This ecosystem allows the skills of data scientists to flourish, including in the medicinal area, and is freely shared around the world via the internet (11). In general, less than 25% of health data is publicly available for research (42). In recent years, big data in drug discovery has been created and collected from the biological, chemical, pharmacological, and clinical fields. It may be the case that in the future, this fact will address the challenges in medicine (8).

To date, the 21st century has been an era of big data involving all aspects of human life, including medicine and healthcare. New fields of technology such as genomics, proteomics, metabolomics, and other types of omics technologies have produced a great quantity of data. Digitalization in clinical practice has enabled this amount to grow exponentially (12).

Today, more than ever, healthcare appears to have in hand new tools with a highly promising potential for evidence-based decisions for the benefit of the patient. AI as well as IoT offer significant advantages. In combination with modern technology such as “smart” nanodevices, they can move clinical practice one step forward.

Nanotechnology is an emerging field that gives new hope for the treatment of various disorders, such as CNS diseases. Nanotheranostics offer targeted and controlled release, which is an integrated approach customized to the anatomy of each patient. They also facilitate the profiling of disease and medicine, as well as a deeper understanding of the relationship between host and disease. All this, combined with the high-quality data provided by nanotechnology applications (e.g., nano-biosensors), opens new avenues for faster and more accurate diagnosis as well as safer treatment.

As mentioned above, ANN can have multiple applications, from supporting clinical results for bioequivalence studies to ensuring CM to appropriately monitor drug shortages, a critical topic in the current pharma business. In addition, therapeutic effects of ANN may range from conditions such as Sjögren’s disease (19) to becoming an ordinary tool in modern dentistry shortly being implemented in fields like Restorative Dentistry, Endodontics, Orthodontics, Dental Surgery, and Periodontology (43).

Various utilizations of RWD range from patients viewing their information, e.g., in a mobile app to increase awareness of their condition, to physicians viewing patient data and having an overview of patient groups or isolated patients to monitor progress or be informed, e.g., of an upcoming epileptic seizure. As another application, AI messaging programs can be safely used by healthcare facilities or healthcare professionals to facilitate patient care (17).

AI in healthcare has the potential to lead to significant improvements in achieving the goals of providing real-time, better personalized and population medicine at lower costs (40). The integration of AI into the healthcare system differentiates the traditional roles of healthcare professionals and creates new opportunities for better healthcare results in terms of quality, safety, and efficacy (6). It is a compelling vision that can lead to significant improvements to achieve the goals of providing more personalized and population-based medicine in real-time at a lower cost. AI can be applied at every step of a pharmaceutical product’s lifecycle. The use of automated algorithms can be implemented for various tasks that previously relied on human intelligence. In recent years, it has redefined how scientists identify new drug targets, perform drug repositioning and repurposing, generate novel molecules, conduct clinical trials, and perform all other activities that constitute the medicinal product lifecycle (9).

The technological evolutions have also had a sounding effect on clinical research. Atomic is an AI tool, developed by Amgen, that can scan internal and public data to identify and rank sites and physicians based on previous performance in recruiting patients for trials, reducing the time of patients’ enrolment by up to half. It is currently being used for conditions including cancer and cardiovascular disease and aims to be used for most studies in the future. It is expected to save 2 years of the decade or more it typically takes to develop a drug. Bayer’s plans include the use of real-world patient data to generate an external control arm that aims to eliminate the need for a placebo. Especially for pediatric trials in cases of rare conditions, ethical concerns could be raised if trial participants were given a placebo when there are no proven treatments available. Anonymized RWD on pediatric patients could be used instead. AI speeds up the process of finding real-world patients by mining electronic patient data drastically (42). The Healthentia platform is an e-clinical solution that collects clinical results from medical and IoT devices and provides advanced services via the use of AI. Thus, virtual guidance is offered with minimal or zero human intervention. This allows healthcare professionals to remotely monitor the processes (17).

Precision medicine is a state-of-the-art, powerful development that has the potential to transform the traditional symptom-driven practice into individual, personalized, and earlier interventions using advanced diagnostics and tailoring better and more economically personalized treatments. Over the years, biotechnology has shown a great evolution. In combination with the development of AI, advances have been achieved in fields such as cancer, neural diseases, and cardiovascular diseases. Tailor-made individualization of treatment has a positive effect in the case, e.g., of trastuzumab for HER2-positive breast cancer. In combination with the use of DL, early diagnosis and therapeutic approaches have been achieved in several other diseases, such as nodular BCC, dermal nevus, seborrheic keratosis in dermatopathology, diabetic retinopathy, type 2 diabetes subgroups, cytological abnormalities in females, cardiac anomalies, and congestive heart failure (40).

As innovation in science and technology rapidly evolves, CRISPR (Clustered Regularly Interspaced Short Palindromic Repeats) is an indisputable breakthrough and a Nobel Prize nominee. CRISPR is a gene-editing tool that is heavily based on AI and ML. ML helps in the prediction of target effects in CRISPR tools that target RNA instead of DNA, broadening the tool’s therapeutic scope. An algorithm predicts protein structure based on smaller CRISPR protein “scalpels” that make genetic snippets more precise. Searches in extensive databases of genetic material, from various sources, from Antarctic shores to dog saliva, discovered hundreds of potential CRISPR variants in bacteria that are rare but stable and effective for editing human genomes. CRISPR leads science one step further from the genome to the epigenome—a family of mechanisms controlling when genes are turned on or off. This already resulted in regulatory approval for the treatment of sickle cell anemia and beta-thalassemia, two painful blood disorders caused by a single genetic typo that distorts blood cells’ shape and limits their ability to deliver oxygen, in the United Kingdom, the United States, and Europe (44).

CRISPR is the spearhead of efforts that began more than 30 years ago when gene mapping allowed the gene therapy revolution to break out as a new potential for the treatment of genetic disorders and multifactorial conditions, even offering polypeptide hormones or enzymes to treat acquired disease. Cloning a normal human gene was no longer a problem. The development of genetic engineering made the human genome not a mystery anymore. The most advanced technology involved the use of viruses, which had been engineered for safety and to accept human genes (45). Since then, gene therapy has been used to treat or demonstrate disease stabilization for many patients worldwide. Focusing mainly on infectious and genetic diseases, it has enormous potential to treat a variety of infectious and genetic diseases, and various pertinent medicinal products have been developed; hematopoietic cell products have been gene-modified with retrovirus vectors carrying therapeutic transgenes. Under strict medical supervision and through continuous development, including a focus on the benefit/risk ratio, gene therapy has shown massive evolution (46). Furthermore, via a combination of knowledge of the disease pathogenesis with the necessary temporal and spatial specificity of gene expression, therapeutic efficacy is achieved. Gene therapy also has potential to offer therapeutic benefits to patients with neurodegenerative diseases in various ways, including direct correction of pathogenic mechanisms, neuroprotection, neurorestoration, and symptom control (47). Probably gene therapy is a history in the making and is in the beginning.

It may be the case that in 30–50 years, novel, innovative, and highly effective treatment modalities for socially dangerous infections such as hepatitis C and HIV will have been discovered. Early diagnostics, using technologies such as updated lines of tumor markers and IoT monitoring technologies, and mass prevention will be the main strategies in oncology. Technological progress may also affect the treatment of depression, obsessive-compulsive disorder, and schizophrenia-like psychoses, probably along with the introduction of new psychotherapeutic modalities. While prevention is still under development, science may progress to a “magic pill,” like CRISPR technology, to keep us forever young. It is also an undoubtful wish that high-quality healthcare services will no longer be available only to those who can pay for them but to all who are in need (48).

Having said that, one major issue that should be addressed is ensuring a secure and ethical data-sharing culture. As big data brings big challenges, the key point is appropriate data sharing while fully respecting data privacy and, in parallel, maintaining the scientific utility of the data. Patients need to fully understand how their data will be used and by whom. Both legal and ethical considerations appear. For example, secondary use of data for research raises ethical issues that require identification, examination, and guidance. Probably a topic of data ethics is raised that may be defined as “studies and evaluates moral problems related to data (including generation, recording, curation, processing, dissemination, sharing, and use), algorithms (including AI, artificial agents, ML, and robots), and corresponding practices (including responsible innovation, programming, hacking, and professional codes), to formulate and support morally good solutions (e.g., right conduct or right values).” Additional challenges are data “ownership” (especially in a commercialized data world), trust, bias, group-level ethical harms, measures of accountability, the distinction between harm to data subjects resulting from the respective academic and commercial uses of big data, providing feedback to patients on unanticipated incidental findings, and data anonymization as a mechanism to share datasets, which removes the need to request the consent of the data subject. The European Data Protection Act is a starting step, but more needs to be done in terms of interpretation and implementation (29).

## 5 Conclusion

Nowadays, it is more than obvious that we are living in exciting times of rapid change in the pharma ecosystem. New promising technologies offer more options for better, safer, and more effective treatments, developed faster and with a more solid, data-driven and evidence-concrete approach to be brought to the patient. In challenging areas such as oncology and rare diseases, as well as in other fields such as cardiovascular diseases, therapeutic solutions are even more feasible to seek, moving toward the gap of unmet medical need. The business environment has been constantly undergoing changes, and new ways of doing business have appeared. It seems that with the growing increase in patient awareness and engagement, more than ever, these vast changes are going to conclude in augmenting benefits for the patient.

## 6 Limitations

This study was not based on an exhaustive review of literature, but on literature that was considered important by the author for the current and future situation. Thus, its approach may be considered subjective. A further comprehensive review of the literature could provide a more global overview.

## Author contributions

GA: Writing – original draft, Writing – review & editing.
